# Role of Kir6.2 subunits of ATP-sensitive potassium channels in endotoxemia-induced cardiac dysfunction

**DOI:** 10.1186/1475-2840-12-75

**Published:** 2013-05-09

**Authors:** Zhong-Wei Yang, Ji-Kuai Chen, Min Ni, Ting Zhao, Ya-Ping Deng, Xia Tao, Guo-Jun Jiang, Fu-Ming Shen

**Affiliations:** 1 Department of Pharmacology, School of Pharmacy, Second Military Medical University, Shanghai 200433, China; 2 Department of Pharmacy, Xiaoshan Hospital, Hangzhou, Zhejiang 311202, China; 3 Department of Pharmacy, Changzheng Hospital, Second Military Medical University, Shanghai 200003, China

**Keywords:** Endotoxemia, Cardiac dysfunction, Kir6.2 subunits

## Abstract

**Background:**

Cardiac dysfunction is well-described in endotoxemia and diagnosed in up to 60% of patients with endotoxic shock. ATP-sensitive potassium (K_ATP_) channels are critical to cardiac function. This study investigates the role of Kir6.2 subunits of K_ATP_ channels on cardiac dysfunction in lipopolysaccharide (LPS)-induced endotoxemia.

**Methods:**

Kir6.2 subunits knockout (Kir6.2^−/−^) and wild-type (WT) mice were injected with LPS to induce endotoxemia. Cardiac function was monitored by echocardiography. Left ventricles were taken for microscopy (both light and electron) and TUNEL examination. Serum lactate dehydrogenase (LDH) and creatine kinase (CK) activities, and tumor necrosis factor-α (TNF-α) levels in both serum and left ventricular tissues were determined.

**Results:**

Compared to WT, Kir6.2^−/−^ mice showed significantly declined cardiac function 360 min after LPS administration, aggravated myocardial damage and elevated serum LDH and CK activities. Apoptotic cells were obviously increased in heart tissues from Kir6.2^−/−^ mice at 90, 180 and 360 min. TNF-α expression in both serum and heart tissues of Kir6.2^−/−^ mice was significantly increased.

**Conclusions:**

We conclude that Kir6.2 subunits are critical in resistance to endotoxemia-induced cardiac dysfunction through reducing myocardial damage by inhibition of apoptosis and inflammation. K_ATP_ channels blockers are extensively used in the treatment of diabetes, their potential role should therefore be considered in the clinic when patients treated with antidiabetic sulfonylureas are complicated by endotoxemia.

## Background

Endotoxemia is one of the leading causes of death in the critically ill patients [1]. Cardiac dysfunction is well-described in endotoxemia and diagnosed in up to 60% of patients with endotoxic shock [2]. More importantly, cardiac dysfunction is one of the key manifestations in clinical endotoxemia that contributes to significant morbidity and mortality in patients in intensive care units [3].

Cardiac dysfunction in endotoxemia is a complex pathophysiological process and the precise mechanisms responsible for myocardial dysfunction in the setting of endotoxemia remain to be unraveled. It is suggested that pro-inflammatory cytokines, such as tumor necrosis factor-α (TNF-α), act directly or indirectly to depress cardiac function resulting in endotoxemia-induced myocardial dysfunction [4,5], and inhibition of the production of inflammatory mediators in the heart attenuates lipopolysaccharide (LPS)-induced myocardial dysfunction [6]. Apoptosis plays an important role in LPS-induced cardiac dysfunction; inhibition of cardiac apoptosis pharmacologically by various treatments prevents LPS-induced myocardial dysfunction [7,8]. Convincing evidence suggests that myocardial dysfunction is closely related to pro-inflammatory cytokines and cardiac apoptosis.

The ATP-sensitive potassium (K_ATP_) channel is an octameric (4:4) complex of two different types of protein subunit: an inward rectifier potassium channel (Kir6.1 or Kir6.2) and a sulfonylurea receptor (SUR1 or SUR2) [9]. The cardiac K_ATP_ channels are composed of Kir6.2 subunits in combination with SUR2A [10]. Studies suggest that opening K_ATP_ channels prevents activation of inflammation and production of a variety of pro-inflammatory factors in microglial cells [11], and blocks cardiocyte apoptosis in ischemia-reperfusion [12]. K_ATP_ channels are also believed to play an essential role in cardiovascular adaptive response under the challenge of stress [13]. Furthermore, Buckley et al. proposed that K_ATP_ channels opening might actually represent a protective mechanism against cellular damage in endotoxemia [10]. However, the role of Kir6.2 subunits of K_ATP_ channels in endotoxemia-induced cardiac dysfunction is unknown. We hypothesized that the Kir6.2 subunits protects cardiac function through reducing myocardial damage by inhibition of apoptosis and inflammation in endotoxemia. In this work, using Kir6.2^−/−^ mice, the role of Kir6.2 on cardiac dysfunction was investigated in LPS-induced endotoxemia.

## Methods

### Experimental animals

Male mutant mice (20–25 g) lacking Kir6.2-containing K_ATP_ channels were generated by targeted disruption of the gene coding for Kir6.2 in the Model Animal Research Center of Nanjing University (AAALAC accredited). Wild-type (WT) littermates were used as controls. Knockout of the Kir6.2 gene was confirmed by RT-PCR (Additional files 1, 2 and 3: Figure S1-S3). Animals were housed under conditions of controlled temperature (22-24°C), 12-h light and 12-h dark cycles (8:00–20:00 light, 20:00–8:00 dark), with free access to food and tap water. All the animals used in this work received humane care in compliance with the institutional animal care guidelines and the Guide for Care and Use of Laboratory Animals published by the National Institutes of Health.

### Endotoxemia model

The endotoxemia was modeled by administration of LPS (Escherichia coli, 0111:B4, Sigma Chemical Co.) referred to previous studies [14,15]. Briefly, LPS was dissolved in sterile saline to a concentration of 1.0 mg/ml. Mice (both WT and Kir6.2^−/−^) were injected with LPS 20 mg/kg intraperitoneally to induce endotoxemia. Mice that were treated with sterile saline served as control.

### Echocardiography

Echocardiographic assessment of cardiac function in mice was examined as described previously [16]. Mice were lightly anesthetized with isoflurane in 100% oxygen 360 min after LPS injection. Transthoracic echocardiography was performed using a 30 MHz high frequency scanhead (VisualSonics Vevo770, VisualSonics Inc. Toronto, Canada). All measurements were averaged for five consecutive cardiac cycles. Echocardiographic assessment of left ventricular ejection fraction (EF) and fraction of shortening (FS) was performed.

### Light and electron microscopy

Changes of left ventricles were examined morphologically as described previously [17]. Mice were anesthetized with pentobarbital sodium (100 mg/kg). Hearts were removed 90 min, 180 min and 360 min after LPS administration. The left ventricles were immersed in a 4% solution of paraformaldehyde in PBS and were fixed in this solution for 24 h. For light microscopy, tissues were washed, dehydrated in a graded ethanol series and embedded in paraffin. Sections (4 μm) were cut transversely, and then stained with hematoxylin and eosin (HE) for light microscopic investigation at a final magnification of 400. For electron microscopy, tissues were fixed as described above and then postfixed with osmium tetraoxide, dehydrated in a graded ethanol series and embedded in epoxy resin. Samples were sectioned (50 nm), counterstained with uranyl acetate and lead citrate and observed with a Hitachi H-800 Transmission Electron Microscope (Hitachi, Japan).

### TUNEL study

Apoptotic changes of the left ventricle were analyzed by a TUNEL method with a commercial kit (Boehringer Mannheim, Mannheim, Germany). Sections (5 μm thickness) were collected on glass slides coated with poly L-lysine. The nuclear DNA fragmentation of apoptotic cells was labeled *in situ* using the TUNEL method. Briefly the sections were first deparaffinized and treated 15 min with 20 mg/ml proteinase K (Boehringer Mannheim) in 0.1 mol/L Tris–HCl buffer (pH 7.4). They were then treated with 2% hydrogen peroxide for 5 min and incubated with 0.3 U/ml TdT buffer (Life Technologies, Gaithersburg, MD, USA) and 0.04 nmol/ml biotinylated dUTP (Boehringer Mannheim) in TdT buffer at 37°C for 60 min. The sections were incubated for 10 min with 2% bovine serum albumin, followed by 30 min in peroxidase-conjugated streptavidin (DAKO, Carpinteria, CA, USA) diluted 1: 300 with phosphate-buffered saline. Peroxidase activity in the sections was visualized by the addition of 0.025% 3, 3-diaminobenzidine tetrahydrochloride in 0.05 mol/L Tris–HCl buffer (pH 7.4) solution containing 0.01% hydrogen peroxide for 5 min. Omission of the TdT enzyme in the TUNEL reaction was used as a negative control and resulted in no staining. Tonsil tissue was used as a positive control. Apoptosis was evaluated by computer-assisted image analysis system (LEICA QUIPS, LEICA Imaging Systems LTD, England) and the results were calculated as the number of positive-staining nuclei per 1,000 cells. For these counts, 2,000 cells were randomly selected from each specimen [18].

### CK and LDH analysis

Mice were anesthetized with pentobarbital sodium (100 mg/kg), blood samples were collected 360 min after LPS administration and stored at 4°C for 24 h. Then the samples were centrifuged at 3,000 g for 10 min at 4°C, and the serum was collected. Serum CK and LDH activities were measured by using commercial kits (Shanghai Changzheng Biotech Ltd, China).

### Enzyme linked immunosorbent assay

Blood samples were collected 90, 180 and 360 min after LPS administration, and centrifuged at 3,000 g for 10 min at 4°C to collect serum. The serum was kept at −80°C until analyzed. The levels of TNF-α was measured with commercial ELISA kits (R&D Systems, Minneapolis, MN, USA).

Left ventricles were harvested 360 min after LPS administration and washed three times in PBS, homogenized, centrifuged at 11,000 g at 4°C for 15 min, and the supernatant was obtained. Protein was quantified with a BCA Protein Assay Kit (Tiangen, Beijing, China) and then the levels of TNF-α was measured with commercial ELISA kits (R&D Systems, Minneapolis, MN, USA).

### Western blot analysis

Protein extraction and concentration determination were the same as described in ELISA. Samples of about 30 μg were run on 10% SDS-PAGE. The proteins were then electrotransferred to nitrocellulose filter membranes. The membranes were incubated in PBS containing 5% non-fat dry milk for 4 h at 25°C. The blots were then incubated for 2 h at 25°C with primary antibodies for Kir6.1 (1:200; Alomone Labs Ltd, Israel), and then incubated with IRDye 800CW-conjugated goat anti-rabbit secondary antibody (1:5,000; Rockland, USA) for 1 hour at 25°C. The infrared fluorescence image was obtained using Odyssey infrared imaging system (Li-Cor Bioscience, Lincoln, NE), and the band were quantified by Image J software (NIH, USA).

### Statistical analysis

Values are expressed as mean ± SEM. Differences between two groups were evaluated by Student’s unpaired t-test. *P*<0.05 was considered statistically significant.

## Results

### Kir6.1 subunits keeps unchanged in heart tissues of Kir6.2^−/−^ mice

**Figure 1:**
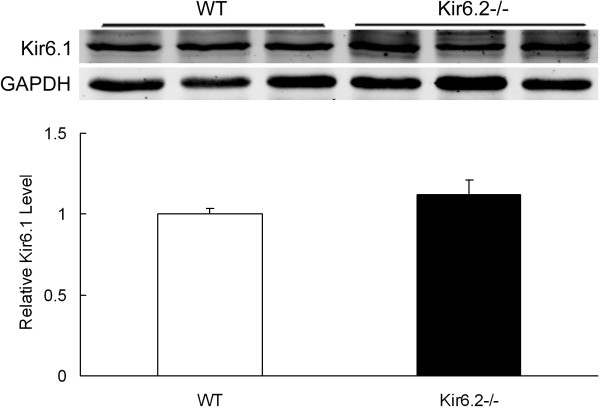
**Unchanged Kir6.1 subunits expression in heart tissues in Kir6.2**^**−/− **^**mice.** Data are expressed as means ± SEM (n=3 per group).

To examine whether the expression of Kir6.1 subunits of K_ATP_ channels were compensatory altered by genetic ablation of Kir6.2, we compared the expression of Kir6.1 subunits in the left ventricular tissues between WT and Kir6.2^−/−^ mice, and found that levels of Kir6.1 subunits were similar between the two groups (1.1 ± 0.09 *vs* 1.0 ± 0.03, n=3, Figure 1).

### Kir6.2^−/−^ deteriorates cardiac function

**Figure 2:**
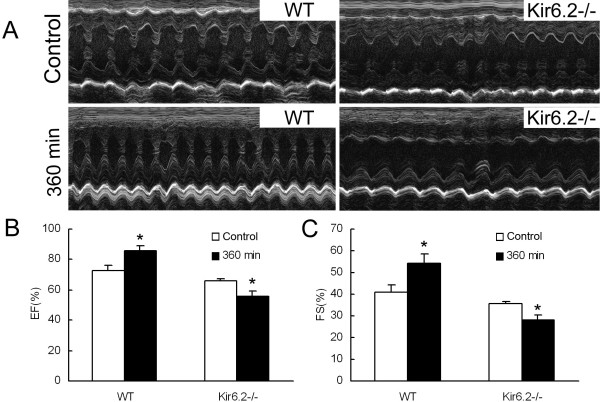
**Lack of Kir6.2 subunits deteriorates cardiac function in mice following LPS treatment (A, representative echocardiograms).** Unlike WT mice, Kir6.2^−/−^ mice failed to augment cardiac performance 360 min after LPS administration, and progressed to significant depression of ventricular function as determined by left ventricular ejection fraction (EF, **B**) and fraction of shortening (FS, **C**). *P<0.05 compared with Control (unpaired *t* test). Data are expressed as means ± SEM (n=10 per group).

As is shown in Figure 2A, the effects of Kir6.2 subunits on cardiac function was assessed with or without LPS treatment (n=10 per group). Echocardiographic examination showed that the baseline values of cardiac function, assessed by EF and FS, were similar between WT and Kir6.2^−/−^ mice though the values of Kir6.2^−/−^ mice were slightly lower. LPS treatment caused significant increase of both EF (86 ± 3 *vs* 73 ± 4 percent, *P*<0.05, Figure 2B) and FS (55 ± 4 *vs* 41 ± 3 percent, *P*<0.05, Figure 2C) when compared with the baseline value in WT mice. However, LPS treatment resulted in lesser EF (56 ± 3 *vs* 66 ± 2 percent, *P*<0.05, Figure 2B) and FS (28 ± 2 *vs* 35 ± 1 percent, *P*<0.05, Figure 2C) in Kir6.2^−/−^ mice.

### Kir6.2^−/−^ aggravates myocardial damage

**Figure 3:**
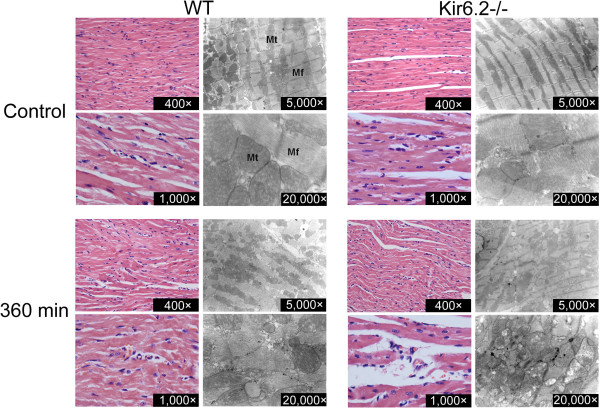
**Morphological analysis of myocardial damage in WT and Kir6.2**^**−/− **^**mice 360 min after LPS administration.** Representative light and electron micrographs of the left ventricular tissues indicate that LPS-induced endotoxemia caused more severe myocardial damage in Kir6.2^−/−^ mice than in WT mice (n = 6 per group). Mt, mitochondria; Mf, myofiber.

To examine the role of Kir6.2 in the pathologic process of myocardial impairment during endotoxemia, histological analysis were performed 360 min after LPS challenge (n=6 in each group). Morphological examination displayed no significant differences in heart tissues of WT and Kir6.2^−/−^ mice under both light and electron microscopy levels. After LPS challenge, hearts from WT mice displayed a mild feature of myocardial damage, including irregular arrangement and mild degeneration of cardiocytes and slight rupture of myocardial fibers. Consistent with the results from light microscopy, electron microscopic examination showed similar phenomena. After LPS challenge, the ultrastructure of cardiocytes in WT mice showed disarray with swollen, vagueness of the mitochondrial membrane and amorphous deposit-laden mitochondria. Compared with these changes found in time-matched WT mice, these lesions were considerably more severe in Kir6.2^−/−^ mice (Figure 3).

**Figure 4:**
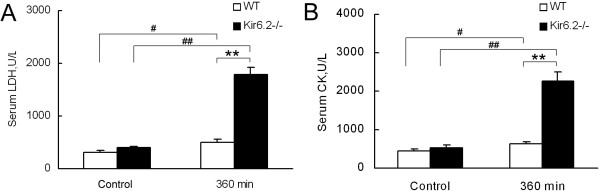
**Serum lactate dehydrogenase (LDH, A) and creatine kinase (CK, B) levels were significantly elevated in Kir6.2**^**−/− **^**mice following LPS treatment.** **P<0.01 compared with WT (unpaired *t* test); ^#^*P*<0.05, ^##^*P*<0.01 compared with Control (unpaired *t* test). Data are expressed as means ± SEM (n=10 per group).

In addition, serum LDH and CK activities, markers for the confirmation of myocardial injury, were determined 360 min after LPS administration (n=10 in each group). Lack of Kir6.2 subunits itself did not influence the levels of LDH and CK when compared with WT mice. LPS challenge induced greater levels of LDH and CK in WT and Kir6.2^−/−^ mice. Furthermore, the level of serum LDH in Kir6.2^−/−^ mice was significantly higher than in WT mice serum (1787 ± 137 U/L *vs* 507 ± 52 U/L, *P*<0.01, Figure 4A). Similarly, serum CK level was also greater in Kir6.2^−/−^ group (2271 ± 218 U/L *vs* 644 ± 49 U/L, *P*<0.01, Figure 4B).

### Kir6.2^−/−^ exacerbates myocardial apoptosis

**Figure 5:**
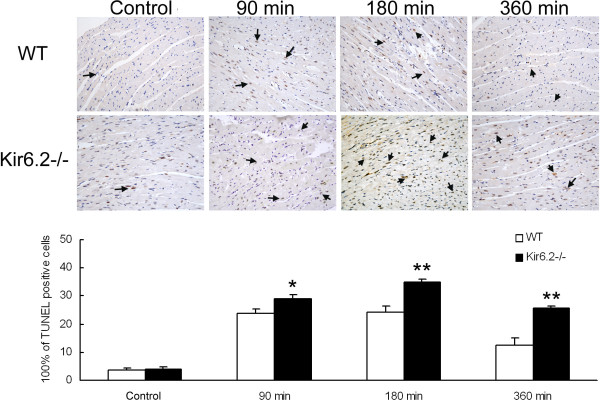
**Lack of Kir6.2 subunits exacerbates myocardial apoptosis in mice at 90, 180 and 360 min after LPS administration (TUNEL × 400).** Arrow indicates TUNEL positive cells. *P<0.05, **P<0.01 compared with WT (unpaired *t* test). Data are expressed as means ± SEM (n=6 per group).

Apoptosis has been recognized as a major contributor in endotoxemia-induced cardiac dysfunction. Thus, the role of Kir6.2 in myocardial apoptosis was examined. Apoptotic cells were only occasionally appeared in sections of left ventricles in either WT or Kir6.2^−/−^ mice. LPS injection caused significant apoptosis in both kinds of mice. However, apoptotic cells were significantly greater at 90 min (29 ± 1.6 *vs* 24 ± 1.5, *P*<0.05, n=6), 180 min (35 ± 1.2 *vs* 24 ± 2.5, *P*<0.01, n=6) and 360 min (26 ± 0.4 *vs* 13 ± 2.5, *P*<0.01, n=6) in Kir6.2^−/−^ mice than time-matched WT ones (Figure 5).

### Kir6.2^−/−^ increases serum and myocardial TNF-α levels

**Figure 6:**
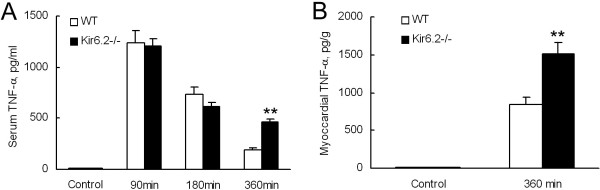
**Serum (A) and myocardial (B) TNF-α levels were significantly elevated in Kir6.2**^**−/− **^**mice following LPS treatment.** Kir6.2^−/−^ mice displayed greater TNF-α level in both serum and myocardial tissues than WT mice 360 min after LPS administration. **P<0.01 compared with WT (unpaired *t* test). Data are expressed as means ± SEM (n=10 per group).

The basic serum and myocardial TNF-α levels in WT mice were undetectable, Kir6.2^−/−^ did not influence these values. Serum TNF-α expression increased rapidly after LPS injection and then tended to decrease. No significant differences were found at 90 min and 180 min between WT and Kir6.2^−/−^ mice. Interestingly, serum TNF-α kept in a higher level in Kir6.2^−/−^ mice at 360 min after LPS treatment when compared with the time-matched WT mice (459 ± 34 pg/ml *vs* 190 ± 21 pg/ml, n=10, *P*<0.01, Figure 6A). LPS injection also induced greater myocardial TNF-α level in Kir6.2^−/−^ mice at 360 min (1510 ± 154 pg/ml *vs* 849 ± 89 pg/ml, n=10, *P*<0.01, Figure 6B).

## Discussion

This study investigated the role of Kir6.2 subunits of K_ATP_ channels in endotoxemia-induced cardiac dysfunction. The main findings are: cardiac dysfunction and myocardial injury were aggravated in Kir6.2^−/−^ mice under LPS-induced endotoxemia. These suggested that Kir6.2 subunits might play a protective role under infectious diseases.

### Kir6.1 subunits expression in the heart

Despite the concept of Kir6.2 and SUR2A subunits as the major molecular component of “cardiac” K_ATP_ channels [10], evidences also support that Kir6.1 is expressed in the heart of mice, and Kir6.1 and Kir6.2 may assemble into functional channel complexes [19,20]. To exclude the possible compensatory action produced by Kir6.1 because of Kir6.2 knockout, expression of Kir6.1 subunits was determined in Kir6.2^−/−^ mice. The similar protein levels of Kir6.1 subunits in the left ventricle between WT and Kir6.2^−/−^ mice suggested that no compensatory increase happened for Kir6.1 subunits in the heart because of Kir6.2 knockout.

### Cardiac protective effect of kir6.2 subunits in endotoxemia

Cardiac dysfunction, diagnosed in up to 60% of patients with endotoxic shock, is a critical determinant of mortality in endotoxic shock [3]. K_ATP_ channel is a kind of molecular sensors that play a critical role in the adaptive response to both physiological and pathological stress [21,22]. Kir6.2 subunit is a major molecular component of “cardiac” K_ATP_ channels. K_ATP_ channels are considered to be a potential target in regulating cardiovascular function in endotoxemia, and the mitochondrial K_ATP_ channels may offer protection against apoptosis in endotoxemia [10]. Thus, the first aim of this work was designed to assess the role of Kir6.2 subunits on cardiac function and cardiac damage under LPS stimulation. Our work found that LPS injection caused a marked decrease of cardiac function in Kir6.2^−/−^ mice, though the cardiac function of WT mice was compensatory increased after LPS treatment. These suggested that lack of Kir6.2 subunits in the heart might not only abolish the compensatory enhancement of cardiac function but also exacerbate cardiac dysfunction in endotoxic shock. Morphological examination showed aggravated myocardial damage in Kir6.2^−/−^ mice, which was also confirmed by the increased LDH and CK activities. To our knowledge, this is the first time to report that lack of Kir6.2 subunits aggravates cardiac dysfunction and myocardial damage during endotoxemia.

### Mechanisms for cardiac protective effect of kir6.2 subunits in endotoxemia

Studies have showed that cardiocyte apoptosis played a mechanistic role in endotoxic myocardial depression [23,24], left ventricular myocyte apoptosis was directly involved in endotoxemia-induced cardiac dysfunction [25], and caspase inhibition prevented heart apoptosis and cardiac dysfunction in endotoxemia [26,27]. In this study, we found that apoptotic cadiocytes were significantly increased at 90, 180 and 360 min after LPS injection in WT and Kir6.2^−/−^ mice. Importantly, the number of apoptotic cadiocytes was greater in Kir6.2^−/−^ mice than time-matched WT ones. Though many factors may contribute to cadiocyte apoptosis during the LPS-produced inflammatory process, our result demonstrated that lack of Kir6.2 subunits increased endotoxemia-induced apoptotic cadiocytes. These suggested that inhibition of apoptosis might be one of the cardioprotective mechanisms mediated by Kir6.2 subunits in endotoxemia.

Inflammatory response has been considered as a pivotal factor in endotoxemia-induced cardiac dysfunction. Levels of various pro-inflammatory cytokines, such as TNF-α, were considered directly or indirectly contributing to cardiac dysfunction [4]. Carlson et al. demonstrated that caspase activation induced by TNF-α mediated endotoxin-related cardiac dysfunction [28]. These suggested that TNF-α signaling played a significant role on cardiac dysfunction in endotoxemia. In this study, we found that both serum and myocardial TNF-α were significantly increased in Kir6.2^−/−^ mice 360 min after LPS injection when compared with WT mice. Mitogen-activated protein kinases (MAPKs), downstream signal proteins of K_ATP_ channels, are considered as molecular targets for anti-inflammatory therapy [29]. Previous study demonstrated that opening of K_ATP_ channels prevented production of TNF-α in microglial cells via inhibition of MAPKs pathways [11]. These results, together with our findings, indicated that inhibition the production of pro-inflammatory cytokines might be another cardioprotective mechanism mediated by Kir6.2 subunits in endotoxemia through MAPKs pathways.

Recently it was found that there was a redistribution of coronary blood flow in the left ventricle during endotoxin shock in pigs, and suggested that ischemic insults might underlie, at least in part, the cardiac dysfunction during endotoxemia [30]. Indeed, hearts of Kir6.2^−/−^ mice lacked the protective effect of ischemic preconditioning [31]. We postulate that myocardial ischemia might be more serious in Kir6.2^−/−^ mice than WT ones during endotoxemia, which might be another possible mechanism responsible for cardioprotective effect mediated by Kir6.2 subunits in endotoxemia. Further study is needed to demonstrate this hypothesis.

### Clinical perspective

Given the issues raised above, it was hoped that Kir6.2 subunits played a vital role in protecting against endotoxemia-induced cardiac dysfunction. Sulfonylureas, a kind of K_ATP_ channels blockers, are extensively used for most patients with type 2 diabetes [32,33]. This suggests that K_ATP_ channels in patients are blocked when drugs lack tissue specificity. Importantly, an increase of plasma glucose may reduce cardiac K_ATP_ channels gene expression to result in cardiac dysfunction observed in diabetic rats [34]. Type 2 diabetes is associated with a high risk of acquiring infectious diseases and developing endotoxemia [35]. Thus, the present study has important implications in the clinic with regard to the relationship between sulfonylureas treatment and heart function for diabetic patients suffering from endotoxemia [36].

### Study limitations

Our work is focused on the in vivo characteristics of Kir6.2 subunits in endotoxemia-induced cardiac dysfunction. To understand the mechanism of Kir6.2 subunits in endotoxemia-induced cardiac dysfunction, further studies with isolated cardiocytes from Kir6.2^−/−^ mice under LPS stimulation is needed, which may offer direct evidence for the protective effects of Kir6.2 subunits under endotoxemia. Besides, the influence of sulfonylureas treatment on cardiac function in diabetic mice under LPS endotoxemia also deserved further investigation.

## Conclusions

That loss of Kir6.2 subunits aggravates endotoxemia-induced cardiac dysfunction is suggestive of a previously unrecognized biological role of this subunit in regulating the cardiac function, and Kir6.2 subunits of K_ATP_ channels may play an important role in resistance to endotoxemia-induced cardiac dysfunction.

## Competing interests

The authors declare that they have no competing interests.

## Authors’ contributions

ZWY and JKC designed and executed the experiments, interpreted data, and wrote the manuscript. MN, TZ, YPD and XT performed molecular biology experiment and animal experiment. GJJ and FMS conceived the study, and participated in its design and helped to draft the manuscript. All authors read and approved the final manuscript.

## Supplementary Material

Additional file 1: Figure S1 Kir6.2 gene expression in Kir6.2^-/-^ mice. Total RNA was prepared from freshly isolated left ventricle of either wild-type or Kir6.2-/- mice. (Kir6.2 primer: sense, TCCAACAGCCCGCTCTAC; antisense, GATGGGGACAAAACGCTG). Click here for file

Additional file 2: Figure S2 Unchanged SUR2 expression in the left ventricle of Kir6.2^-/-^ mice. Click here for file

Additional file 3: Figure S3 Representative ECG from WT and Kir6.2^-/-^ mice. Click here for file
