# Supplementary figures and images for: Role of Kir6.2 subunits of ATP-sensitive potassium channels in endotoxemia-induced cardiac dysfunction
Source: Cardiovasc Diabetol. 2013 May 9;12:75. doi: 10.1186/1475-2840-12-75 (PMC3654940; doi:10.1186/1475-2840-12-75)

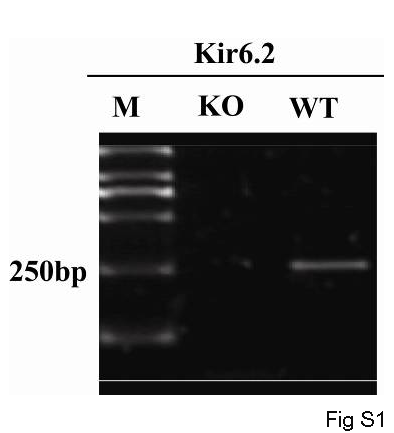

Supplement: Additional file 1: Figure S1 — Kir6.2 gene expression in Kir6.2-/- mice. Total RNA was prepared from freshly isolated left ventricle of either wild-type or Kir6.2-/- mice. (Kir6.2 primer: sense, TCCAACAGCCCGCTCTAC; antisense, GATGGGGACAAAACGCTG). [file 1475-2840-12-75-S1.tiff]

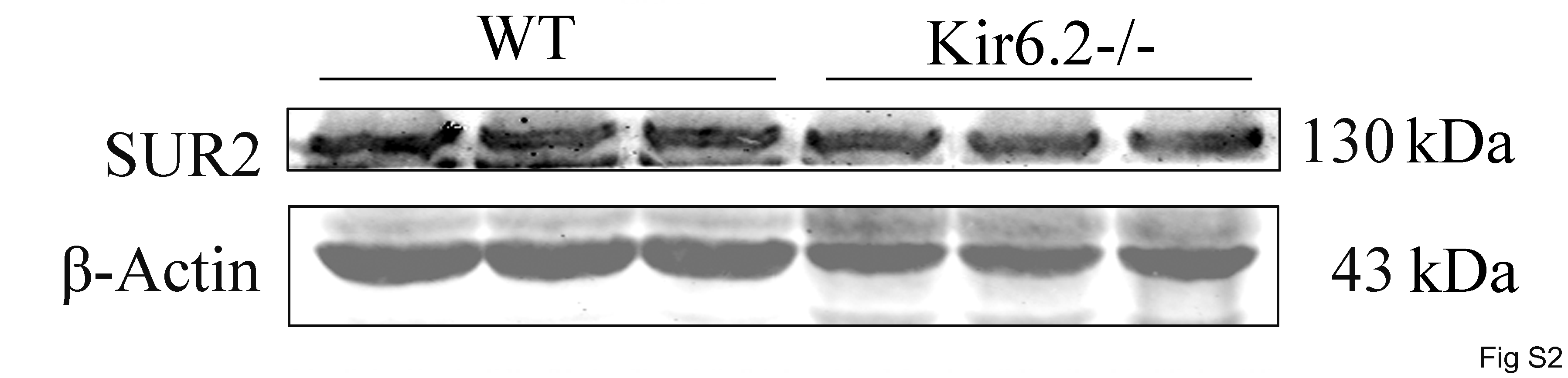

Supplement: Additional file 2: Figure S2 — Unchanged SUR2 expression in the left ventricle of Kir6.2-/- mice. [file 1475-2840-12-75-S2.tiff]

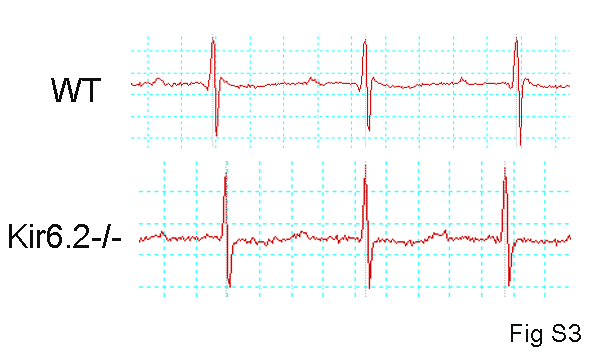

Supplement: Additional file 3: Figure S3 — Representative ECG from WT and Kir6.2-/- mice. [file 1475-2840-12-75-S3.tiff]
